# Synthesis, structure, and properties of carbon/carbon composites artificial rib for chest wall reconstruction

**DOI:** 10.1038/s41598-021-90951-8

**Published:** 2021-05-28

**Authors:** Zhoujian Tan, Xiang Zhang, Jianming Ruan, Jiqiao Liao, Fenglei Yu, Lihong Xia, Bin Wang, Chaoping Liang

**Affiliations:** 1 State Key Laboratory for Powder Metallurgy, Central South University, Changsha, 410083 Hunan People’s Republic of China; 2 Hunan Tankang Biotech Co., LTD., Changsha, 410083 Hunan People’s Republic of China; 3 Department of Thoracic Surgery, The Second Xiangya Hospital, Central South University, Changsha, 410011 Hunan People’s Republic of China; 4 College of Chemistry and Chemical Engineering, Central South University, Changsha, 410083 Hunan People’s Republic of China

**Keywords:** Biomaterials, Composites

## Abstract

In this work, braided carbon fiber reinforced carbon matrix composites (3D-C/C composites) are prepared by chemical vapor infiltration process. Their composite structure, mechanical properties, biocompatibility, and in vivo experiments are investigated and compared with those of traditional 2.5D-C/C composites and titanium alloys TC4. The results show that 3D-C/C composites are composed of reinforced braided carbon fiber bundles and pyrolytic carbon matrix and provide 51% open pores with a size larger than 100 μm for tissue adhesion and growth. The Young’s modulus of 3D-C/C composites is about 5 GPa, much smaller than those of 2.5D-C/C composites and TC4, while close to the autogenous bone. 3D-C/C composites have a higher tensile strength (167 MPa) and larger elongation (5.0%) than 2.5D-C/C composites (81 MPa and 0.7%), and do not show obvious degradation after 1 × 10^6^ cyclic tensile loading. The 3D-C/C composites display good biocompatibility and have almost no artifacts on CT imaging. The in vivo experiment reveals that 3D-C/C composites artificial ribs implanted in dogs do not show displacement or fracture in 1 year, and there are no obvious proliferation and inflammation in the soft tissues around 3D-C/C composites implant. Our findings demonstrate that 3D-C/C composites are suitable for chest wall reconstruction and present great potentials in artificial bones.

## Introduction

Bone defects of chest wall are commonly observed after clinical treatment and intervention such as tumor, infection and radiation injury, and direct damage caused by traumatic factors. If there are more than three adjacent ribs broken or concurrent spinal injuries within large-scale chest wall defect (over 6 × 6 cm defect area), chest wall bone reconstruction must be performed^1^. Various materials were utilized in the chest wall bone reconstruction. For instance, in 1950, Beardsley first used tantalum plate to repair chest wall defects^2^. In the 1980s, bone cement polymethylmethacrylate (PMMA) was applied in chest wall reconstruction. Later, stainless-steel plate and titanium alloy were adopted for chest wall reconstruction^3^.

However, these commercialized materials have some drawbacks in terms of surgical recovery. For example, the hand-made PMMA implants could easily induce postoperative soft tissue hematoma and infection due to their low surface flatness and rough edges. Moreover, they are prone to fragmentation and fracture when subjected to the external force^3^. The metal products, like stainless steel and titanium alloys, are generally incompatible to human tissues^4–6^. Firstly, metals have poor bone regeneration ability and poor compatibility with surrounding tissues. It is reported that the probability of postoperative infection after implantation is about 5%^7^. Secondly, the high Young’s modulus (above 100 GPa for TC4) could cause further damage and displacement under external impact^8^. Studies have shown that there is a high incidence of implant-related complications (such as fracture and displacement) (about 44% in which implant fracture accounts for 37% and displacement accounts for 7%) within 1 year after surgery^6^. Thirdly, metals can affect the diagnosis and treatment of subsequent diseases, i.e., magnetic resonance imaging (MRI) examination is almost impossible after steel implantation.

In view of above issues, biological fixation system has been proposed to replace traditional mechanical fixation system. The biological fixation system requires the implant materials have good biocompatibility^9^. For chest wall reconstruction, the materials should have a tensile strength and Young’s modulus ranged from 60 to 160 MPa and 3 to 30 GPa, respectively^10^. They also should have a large contact area with cortical bone to promote bone regeneration.

**Figure 1:**
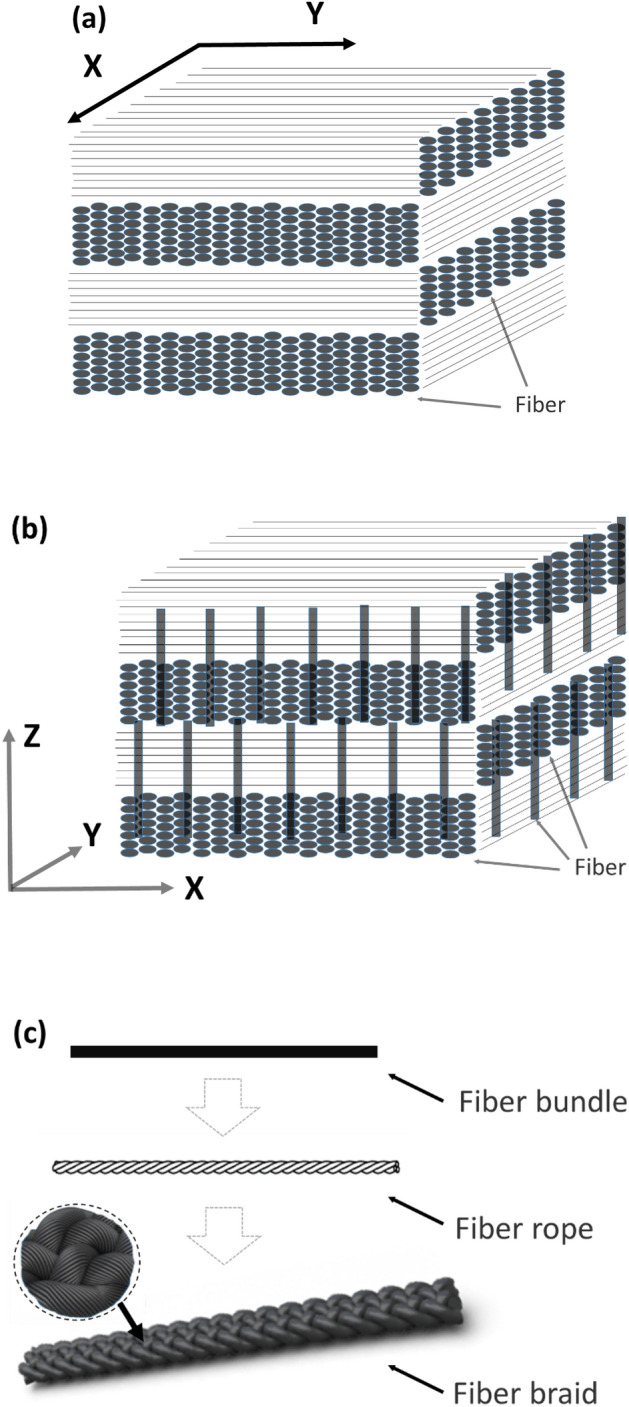
Architecture of preform for C/C composites, (**a**) 2D-C/C composites; (**b**) 2.5D-C/C composites; (**c**) 3D-C/C composites, respectively.

Carbon is known for its excellent biocompatibility^11^, and carbon materials such as carbon fibers, pyrolytic carbon, carbon nanotubes and their composites, are widely used in heart valves, bones, tendons, growth scaffold, tumor drugs, biosensors, etc.^12–17^. Carbon/carbon (C/C) composites with carbon as matrix and carbon fiber and its fabric as reinforcement have the characteristics of light weight, similar Young’s modulus to human bone and strong design ability, which makes them a promising biomaterial in artificial ribs^18,19^. The engineered C/C composites can be classified into two types according to the literature^20–22^. The 2D-C/C composites featured with perpendicular fiber bundles overlayed one by one (Fig. 1a), while the 2.5D-C/C composites have extra dispersed fibers along the vertical direction (Fig. 1b). There are some studies adopt C/C composites as artificial bones and show good biomaterial performance^23–26^. For example, Szabó et al.^23^ used 2D-C/C composites with purity of 99.9% for mandibular reconstruction. Clinical experiments suggested that in the long-term observation, carbon plate fracture, screw loosening, infection or inflammation around C/C composites implants have not occurred, and C/C composites implants has been successfully replaced by autogenous bone. Wang et al.^24^ prepared 2.5D-C/C composites by chemical vapor deposition to improve biocompatibility and reduce debris release. They found that 2 weeks after implantation in New Zealand white rabbits, the implant was covered with fibroblasts, and there was only slight tissue inflammation. The swelling and edema were significantly reduced in 8 weeks, and there was no obvious inflammatory tissue around the implant in 12 weeks. Moreover, the fragments of the implant in rabbits were limited. Baquey et al.^25^ inserted 2D-C/C composites into the dog’s femoral artery for in vivo experiments and found that in the first hour, the C/C composites induced the aggregation of platelet, while no aggregation of red blood cells and fibrin. Pesáková et al.^26^ showed that 60 days after 2D-C/C composites were implanted in animals, the animal's immune system reacts slightly. However, those engineered 2D or 2.5D-C/C composites are very fragile and unable to sustain high external impact, which limits the widely application in artificial ribs for the chest wall reconstruction.

Up to now, Researchers^27–30^ have mainly focused on the improvement of the composition and surface quality of C/C composites, while played less attentions on the composite structure itself. In this study, carbon fiber reinforced carbon matrix composites were fabricated through a well-designed composite structure (we name it 3D-C/C composites). The newly fabricated 3D-C/C composites were used as artificial ribs for the chest wall reconstruction of large mammals and showed superior performance than the commercial Ti-6Al-4 V (TC4) and traditional 2.5D-C/C composites. The paper was organized as follows. “Materials and methods” described the composite structure and preparation methods of 3D-C/C composites, and the characterization methods of the materials and their biological behaviors. In “Results and discussion”, we compared the material properties and biological performance among commercial TC4, traditional 2D-C/C composites and our 3D-C/C composites. The difference among those materials were discussed and highlighted, and a conclusion was draw in “Conclusions”.

## Materials and methods

### Materials

Two types of C/C composites were prepared in this study. One is braided carbon fiber preform reinforced carbon matrix composites (3D-C/C composites), and the other is 2.5D needle-punched carbon fiber preform reinforced carbon matrix composites (2.5D-C/C composites). Figure 1 shows the architecture of carbon fiber preforms. The 3D braided carbon fiber preform was fabricated with strips of twisting three strands of carbon fiber (Fig. 1c). The 2.5D needled carbon fiber preform was made up of layers of non-woven carbon fiber cloth and short-cut carbon fiber web, which are stacked alternatively and attached to each other by needle-punching in thickness-direction step by step. Layers of non-woven carbon fiber cloth were oriented at 0°/45°/90°/135° (Fig. 1b). The fiber volume fraction of 3D braided and 2.5D needled carbon fiber preforms were about 70% and 30%, respectively. The carbon fiber we used were 12 k polyacrylonitrile-based carbon fiber (Zhongfu Shenying Carbon Fiber Co. Ltd, China). 3D braided and 2.5D needled carbon fiber preforms were densified by isothermal chemical vapor infiltration (ICVI) at the temperature of 1100 °C, using natural gas as carbon source precursor^31^. The carbon content of the prepared 3D-C/C composites and 2.5D-C/C composites is more than 99.9%. Medical titanium alloy, TC4, was purchased from Baoji Litai Nonferrous Metal Co., Ltd, China.

### Microstructure characterization

The Quanta FEG 250 scanning electron microscope (SEM) with applied voltage ranged from 10 to 15 kV was used to analyze the surface and tensile fracture surface of 3D-C/C composites and 2.5D-C/C composites. The Leica DM 4000 M metallographic microscope was chosen to analyze the cross-sectional characteristics of 3D and 2.5D-C/C composites. AutoPore IV 9500 mercury porosimeter was adopted to analyze the void structure of 3D and 2.5D-C/C composites. The Quantum GX PE micro computed tomography (Micro-CT) was used to analyze the topography of 3D and 2.5D-C/C composites with a scan rate of 72 μm/slice. The samples for tests were 65 × 12 × 3.4 mm in dimension. Medical imaging of materials and their animal implants was performed by Siemens somatom force dual source CT scanner, 1 mm/ slice.

### Mechanical tests

Tensile properties of C/C composites were tested by Instron 8802 mechanical testing machine. The samples of 3D-C/C composites for tests were 150 × 12 × 3.4 mm in dimension, the samples of 2.5D-C/C composites for tests were 150 × 10 × 4 mm in dimension. The tension-tension fatigue test conditions are as follows, the stress ratio is 0.1, the frequency is 5 Hz, sine wave, and the cycle is over 1 × 10^–6^ times. Flexural strength of C/C composites was tested by Instron 3369 mechanical testing machine. Impact ductility of C/C composites were tested by the standard of ISO148-1: 2006. The samples of 3D-C/C composites for tests were 55 × 12 × 3.4 mm in dimension, the samples of 2.5D-C/C composites for tests were 55 × 10 × 4 mm in dimension.

### Culture of MG-63 cells

Human osteoblast-like cells MG-63 (purchased from the Center of Cell Resource, Shanghai Institutes for Biological Science, China) were cultured in Dulbecco modified essential medium (DMEM) cell culture medium supplemented with 10% (v/v) fetal bovine serum (Gibico), 100 U/ml penicillin, and 100 µg/ml streptomycin (Sangon Biotech) at 37 °C in a humidified incubator (BB15, Thermo Scientific, USA) with an atmosphere of 5% CO_2_.

### Cytocompatibility experiment

Cytocompatibility testing of the samples were performed using an indirect method adapted from ISO 10,993:5 and Alamar Blue assay. Extracts were obtained by immersing specimens in DMEM growth medium at a ratio of 1 g:10 mL for 24 h at 37 °C in incubator. After 24 h, the individual medium extracts form each group of specimens were collected for further use.

Cells were harvested and adjusted to a final concentration of 6 × 10^3^ cell/mL. Then, 500 µL cell suspensions were piped in each well of 48-well plate (Greiner bio-one, Germany). The plate was then incubated at 37 °C in CO_2_ incubator.

Cell proliferation were determined by Alamar Blue assay according to the protocol in 1, 3, and 5 days. Briefly, 500 µL of Alamar Blue reacting buffer (Alamar Blue reagent/cell culture medium, 1/20 v/v) was added to each well and incubated at humidified CO_2_ incubator. After 6 h incubation, 100 µL solution was transferred into a new 96-well ELISA plated. The absorbance of solution was measured using a microplate reader (MK3; Thermo) at 570 and 630 nm. The reduction was calculated according to the manufacturer instruction. After testing at the specific time period, the remaining reacting buffer in the 48-well plate was removed and the corresponding extract of specimens was then replaced for further culture.

### In vitro cell seeding

Samples (8 × 8 × 3 mm) were cleaned with ethanol and dried at 60 °C for 30 min. Then they were sterilized by autoclaving at 120 °C for 30 min. After washing twice with phosphate buffer saline (PBS), the sterilized specimens were prewetted in cell culture medium for 24 h in a 24-well plate (Greiner bio-one, Germany). Cells were harvested and adjusted to a final concentration of 5 × 10^3^ cell/mL. Then, 1000 µL cell suspensions were piped onto each sample. The specimens were then incubated at 37 °C in CO_2_ incubator.

### Morphology of MG-63 cells

All collected samples, which were cultured for 1 day, were washed twice with PBS at 37 °C. Samples were pre-fixed with 3% glutaraldehyde for 30 min, and were dehydrated with a serial of dehydration solutions consisting of ethanol. The dehydrated specimens were observed by SEM.

All collected samples, which were cultured for 5 days, rinsed with PBS solution for three times and stained with high-glucose DMEM contained 1 ng/mL fluorescein diacetate solution for 5 min in the dart at room temperature. After staining, the samples were gently washed with PBS solution for three times and observed with fluorescence microscopy (Olympus IX71) immediately.

### In vivo study

The experimental animal was a male adult Chinese garden dog (12 months old, weighing 14 kg). The two ends of the artificial rib were fixed to the stump of the seventh rib on the right side of the dog by cruciate ligament suture, and the reconstruction length was about 5 cm. One year after operation, the artificial rib was removed, and the adhesion between the artificial rib and the surrounding soft tissue was photographed. The soft tissue at the joint of artificial rib and rib and the soft tissue at rib were examined by paraffin section using hematoxylin-eosin (HE) staining to observe the proliferation and inflammatory reaction of soft tissue.

All procedures involving animals were approved by the animal ethics committee of Central South University, People’s Republic of China (No. SYXK 2017-0002). The methods were carried out in accordance with the Guidelines for Care and Use of Laboratory Animals of Central South University, People’s Republic of China. The methods were carried out in accordance with ARRIVE guidelines^32^.

### Statistical analysis

All data were expressed as mean ± standard deviation (SD) and were analyzed using a two-tailed unpaired *t *test (two-group comparison). A value of P less than 0.05 was considered statistically significant.

## Results and discussion

### Microstructure

**Figure 2:**
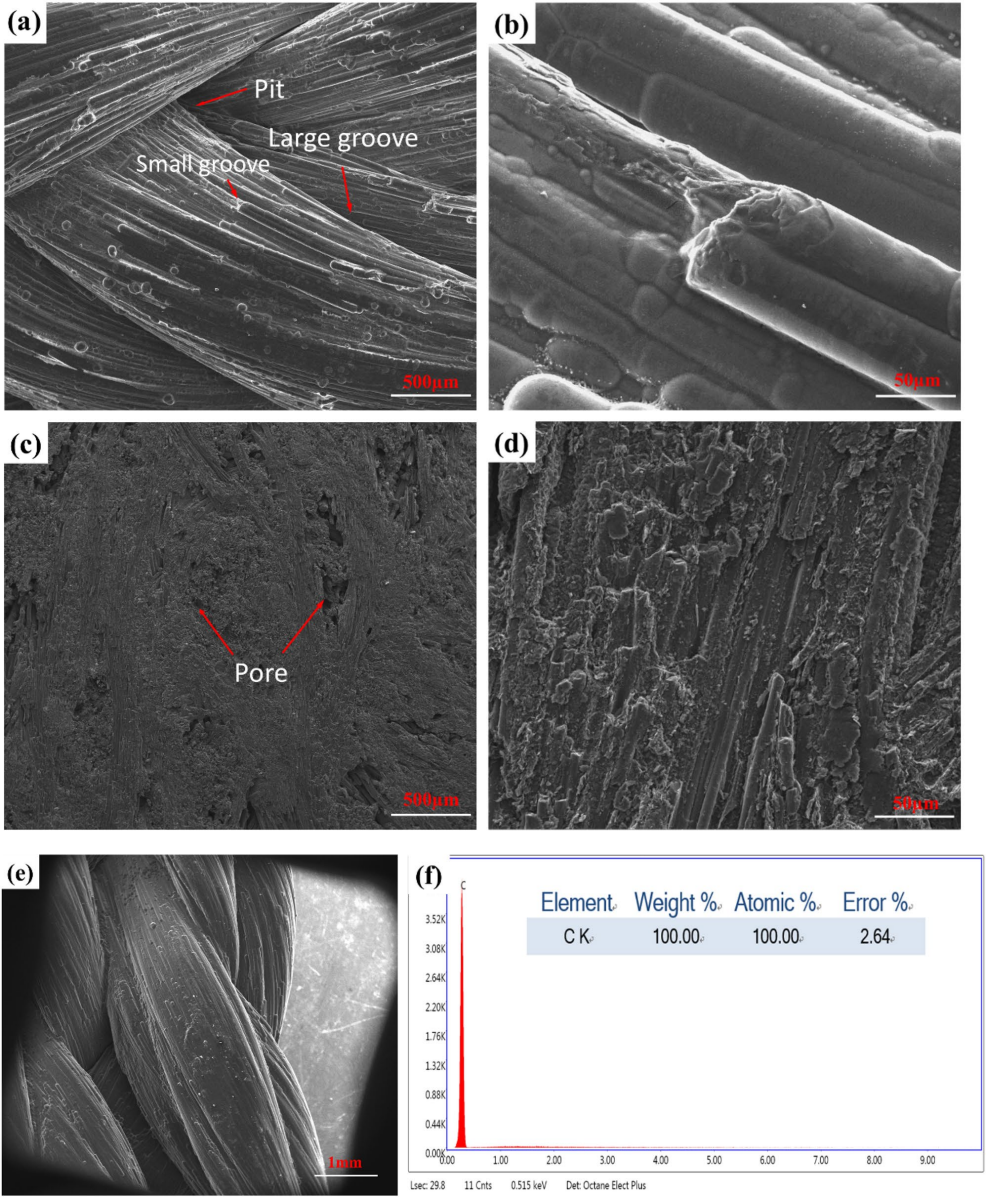
SEM of C/C composites, surface morphology, (**a**), (**b**), (**e**) 3D-C/C composites, (**c**), (**d**) 2.5D-C/C composites; EDS analysis, (**f**) 3D-C/C composites, respectively.

Figure 2 shows the SEM images of the surface morphology of the newly synthesized 3D-C/C composites and traditional 2.5D-C/C composites. The 3D-C/C composites display different features from the traditional 2.5D-C/C composites. As shown in Fig. 2a, carbon fiber braids are orderly arranged according to our composite design in Fig. 1c. Regular pits and grooves forms as result of the crossing and entanglement of different fiber braids, displaying a typical 3D twisted fiber bundles morphology. Specifically, the grooves can be divided into two types, the large one between fiber bundles and small one in fiber bundles. Figure 2b shows the pyrolytic carbon attaches and grows on the fiber braids, implying a 3D-C/C composite structure. The 2.5D-C/C composites, on the other hand, shows a flat surface with irregular pores (Fig. 2c). The zoom-in image (Fig. 2d) shows the parallel fiber arrangement and pyrolytic carbon covers the surface of the nonwoven fiber layer^33^. The micro computed tomography (Micro-CT) further confirms the surface morphology and roughness of 3D and 2.5D-C/C composites. The shape of fiber braids was preserved after infiltration, which leads to a very rough surface on 3D-C/C composites (Supplementary Fig. S1a). On the contrary, 2.5D-C/C composites have a flat surface (Supplementary Fig. S1b). In addition, it could be seen that pores in 3D-C/C composites cut through the sample, while those in 2.5D-C/C composites are on the surface or inside the sample. The rough surface results from large fiber braids (Fig. 2e, Supplementary Fig. S1) could enable the growth of autogenous bone. The energy dispersive spectroscopy (EDS) (Fig. 2f) shows the composites are consisted of almost pure carbon.

**Figure 3:**
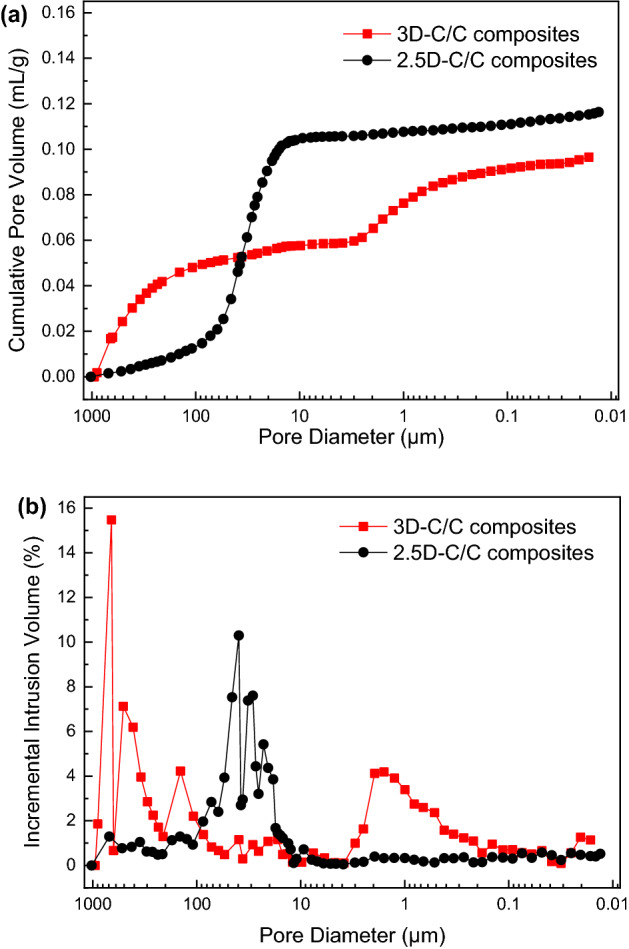
Pore analyses of 3D and 2.5D-C/C composites, (**a**) cumulative pore volume; (**b**) incremental intrusion volume, respectively.

The pore characteristics of both 3D and 2.5D-C/C composites are analyzed by mercury intrusion. Table 1 lists the density and pore characteristics from mercury intrusion. Both 3D and 2.5D-C/C composites have similar density and open porosity. However, the pore size and connectivity are different between 3D and 2.5D-C/C composites. It can be seen from Fig. 3a that the pore size of 2.5D-C/C composites is distributed in a narrow range of 10–100 μm. 3D-C/C composites has two bumps in Fig. 3a, indicating the pores are composed of large (> 100 μm) and small (< 10 μm) pores. This pore size distribution originates from the 3D fiber braids design, in consistent with the small and large grooves observed from SEM images (Fig. 2). The relative percentage of various pore size are shown in Fig. 3b and summarized in Table 1. 79% pores have a size of 10–100 μm for 2.5D-C/C composites, while for 3D-C/C composites 40% and 51% pores are < 10 μm and > 100 μm, respectively. Moreover, the polarized light microscope (Supplementary Fig. S2) and Micro-CT images (Supplementary Fig. S1) show that the majority pores in 3D-C/C composites are open pores, while those in 2.5D-C/C composites are close pores. According to references^34,35^, open pores with pore size ranges from 50 to 300 μm can utilize the growth of bone tissues. We would expect the 3D-C/C composites have better tissue compatibility than traditional 2.5D-C/C composites as a result of more open pores and rougher surface, despite they have similar pore density.

**Table 1:** Pore characteristics of C/C composites from mercury injection.

Type	Bulk density	Open porosity	Percentage of different pore diameter (%)		
	g/cm^3^	%	≤ 10 μm	10–100 μm	≥ 100 μm
3D-C/C composites	1.4	16	40	9	51
2.5D-C/C composites	1.5	18	10	79	11

### Mechanical properties

**Figure 4:**
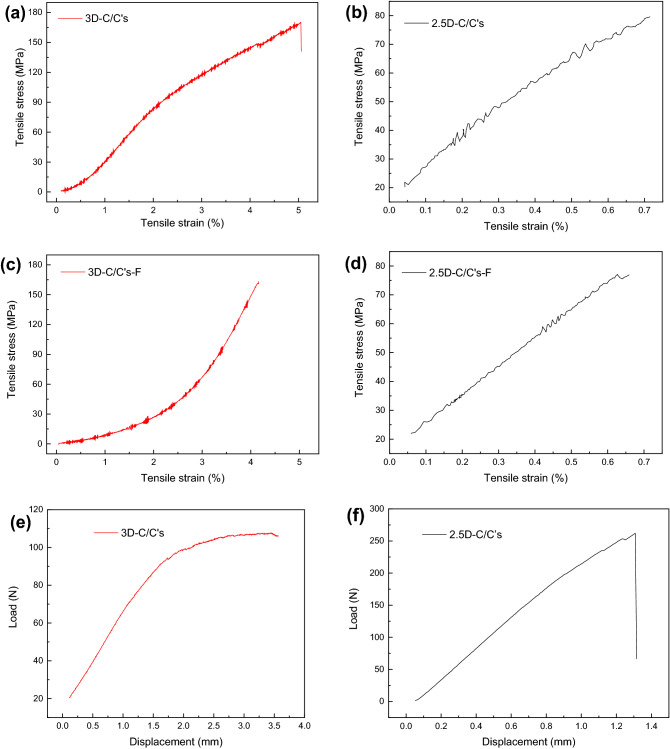
Mechanical properties curve of C/C composites, tensile, (**a**) 3D-C/C composites, (**b**) 2.5D-C/C composites under static condition; (**c**) 3D-C/C composites, (**d**) 2.5D-C/C composites after fatigue; flexural, (**e**) 3D-C/C composites, (**f**) 2.5D-C/C composites, respectively.

The mechanical properties are investigated by tensile, flexural and impact tests. Figure 4 shows the stress–strain curve of 3D and 2.5D-C/C composites. The 3D-C/C composites display a non-linear curve while 2.5D-C/C composites a linear curve for all static, flexural, and fatigue tests in Fig. 4. As showed in Table 2, the elongation of 3D-C/C composites is 5.0% which is significantly larger than that of 2.5D-C/C composites (0.7%). This indicates that 3D-C/C composites are tougher than 2.5D-C/C composites. More importantly, the Young’s modulus of 3D-C/C composites (5 GPa) is smaller than that of 2.5D-C/C composites (12 GPa), while the tensile strength (167 MPa) is larger than that of 2.5D-C/C composites (81 MPa). The flexural strength of 3D-C/C composites (47 MPa) is smaller than that of 2.5D-C/C composites (131 MPa). However, the impact toughness of 3D-C/C composites (> 13.3 J/cm^2^) is much better than that of 2.5D-C/C composites (7.5 J/cm^2^), since the 3D-C/C composites do not fracture after the impact (Supplementary Table S1). The mechanical properties of after tensile fatigue test show similar feature as those of pristine samples, such as the tensile strength and elongation.

**Table 2:** Mechanical properties of C/C composites.

Type	Tensile strength, MPa	Tensile module, GPa	Elongation, %	Flexural strength, MPa	Impact toughness, J/cm^2^
**Static**					
3D-C/C’s	167	5	5.0	47	>13.3
2.5D-C/C’s	81	12	0.7	131	7.5
**After fatigue**					
3D-C/C’s-F	163	9	4.2	–	–
2.5D-C/C’s-F	77	11	0.7	–	–

The fractography of 2.5D and 3D-C/C composites after tensile tests are shown in Supplementary Fig. S3 and Fig. S4, respectively. 2.5D-C/C composites shows complete transverse fracture after failure, while 3D-C/C composites does not break after failure. It can be seen that 3D-C/C composites breaks from single carbon fiber bundle first, while others are just elongated. Both 2.5D and 3D-C/C composites show typical brittle fracture, but it is clear that more fibers are pulled out after fatigue (1 × 10^6^ cyclic tensile loading) for 2.5D-C/C composites than 3D-C/C composites. In addition, the fiber bundles of 3D-C/C composites are more dispersed after fatigue, and pyrolytic carbon still adheres to the outer layer.

The mechanical properties demonstrate that 3D-C/C composites are more suitable for chest wall reconstruction than 2.5D-C/C composites. Firstly, 3D-C/C composites have a smaller Young’s modulus, which is closer to the Young’s modulus (3–30 GPa) of autogenous bone^10^. Secondly, 3D-C/C composites are much tougher than 2.5D-C/C composites, making 3D-C/C composites less likely to disposition and break after external impact. Last but not least, the fracture mode could prevent the suddenly disfunction of artificial bones and may provide opportunity for future bone regeneration.

### In vitro biocompatibility evaluation

**Figure 5:**
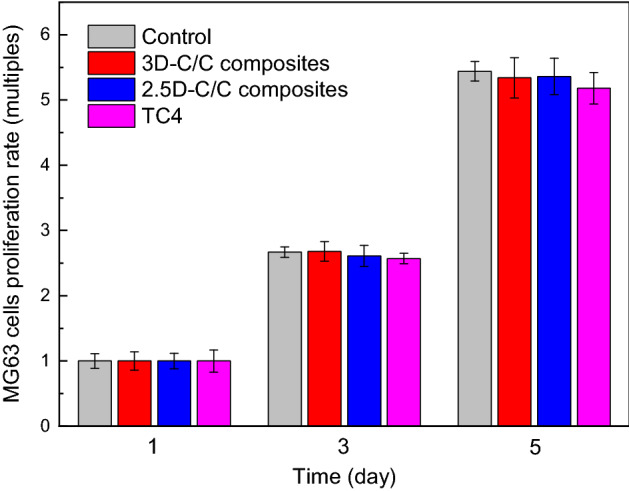
MG-63 cells proliferation rate of 2.5D-C/C composites, 3D-C/C composites, and TC4, the control group is listed as reference.

We turn to studying the biocompatibility of 3D-C/C composites, 2.5D-C/C composites and TC4. Figure 5 shows the proliferation of MG-63 cells on 3D-C/C composites, 2.5D-C/C composites and TC4. It can be seen from this figure that there is no significant difference in cell proliferation rate compared with the control group (P > 0.05) for 3D, 2.5D-C/C composites and TC4. It could be deduced that 3D-C/C composites have better biocompatibility and shows almost no toxicity to cells. Supplementary Fig. S5 and Fig. S6 show the morphology of MG-63 cells growing on different materials. After 1 day growth, cell adhesion has been observed on the surface of the material. It is clear that most of the cells have spread on the material surface, showing typical morphology like fibroblasts, which indicates that MG-63 cells can effectively adhere and spread on three materials. After 5 days growth, the surfaces of the three groups of samples were covered by MG-63 cells, and the cells were closely arranged, leaving only a few gaps between the cells. It could be concluded that MG-63 cells can effectively adhere, spread and proliferate on 3D-C/C composites surface, indicating that it has better cell compatibility.

### Medical imaging

**Figure 6:**
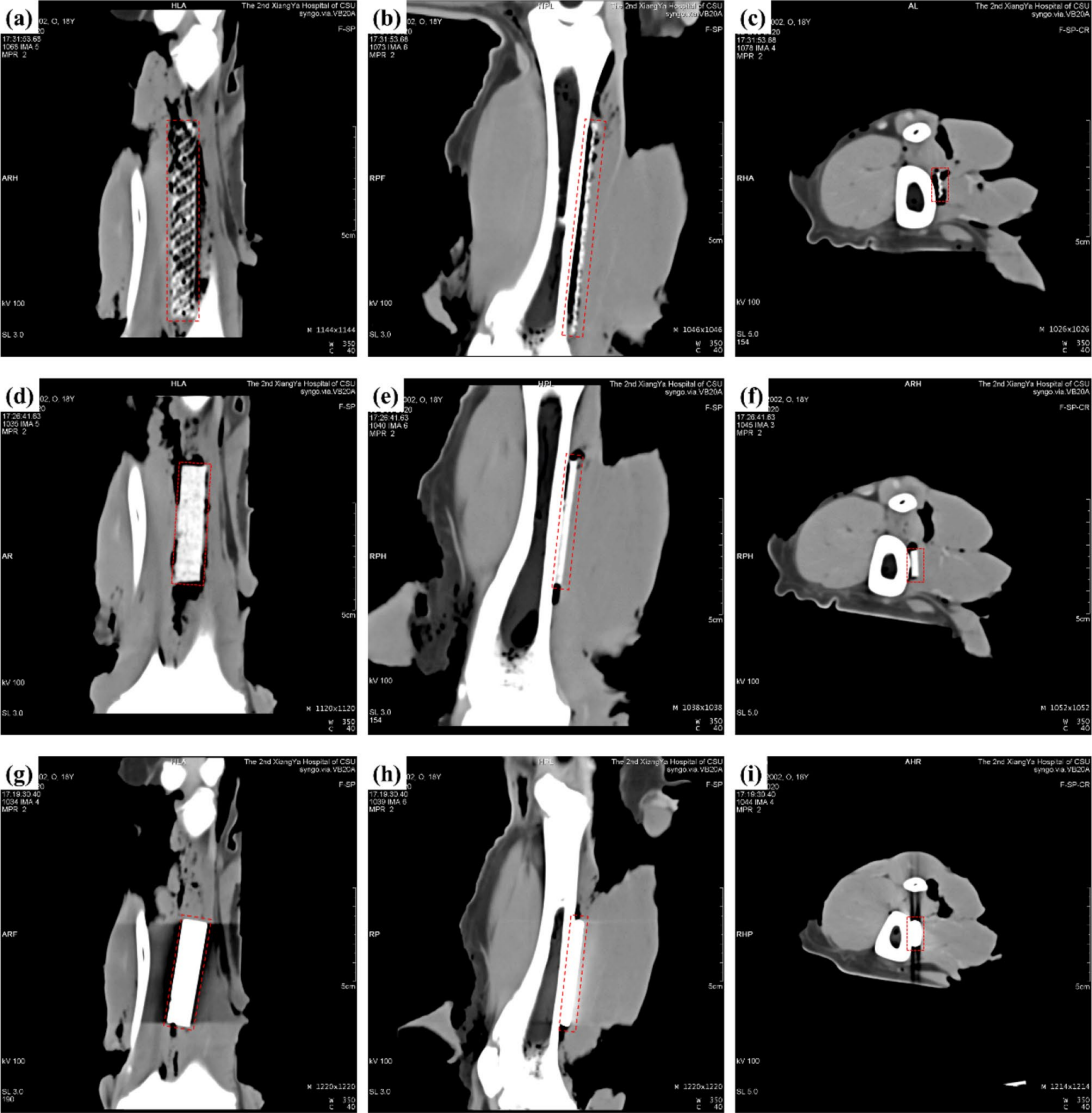
CT images of materials implanted into the shinbone of pig legs (Sample is in the dotted circle), (**a**–**c**) for 3D-C/C composites, 80 × 12 × 3.4 mm in dimension; (**d**–**f**) for 2.5D-C/C composites, 55 × 12 × 3.4 mm in dimension; (**g**–**i**) for TC4, 55 × 12 × 3.4 mm in dimension, respectively.

In order to check the influence of implant materials on the CT images, 3D-C/C composites, 2.5D-C/C composites and TC4 were put into the shinbone of pig legs. Figure 6 shows the three-dimension reconstruction of CT images of 3D-C/C composites, 2.5D-C/C composites and TC4. The periphery of implants in TC4 (Fig. 6g–i) have obvious occlusion scattering images, while 3D-C/C composites (Fig. 6a–c) and 2.5D-C/C composites (Fig. 6d–f) have no scattering, which is consistent with experimental observation in the literature^36,37^. It could be deduced that 3D-C/C composites implants have almost no influence on the medical CT imaging, implying the implants do not affect the diagnosis and treatment of subsequent diseases of patients.

### In vivo biocompatibility evaluation

The in vivo experiments of 3D-C/C composites were performed by implanting an artificial rib into a large mammal dog. Supplementary Fig. S7 is a CT image of 3D-C/C composites artificial rib after implantation. This figure shows that 3D-C/C composites artificial rib is well fixed without displacement and fracture, and the X-ray penetrability of 3D-C/C composites artificial rib is equivalent to that of native bone density, with clear image and no radiation artifacts. The 3D-C/C composites artificial rib was removed 1 year after the chest wall reconstruction operation in the large mammal dogs, and the adhesion of 3D-C/C composites artificial rib to the surrounding soft tissue after implantation was photographed (Supplementary Fig. S8a,b). It can be seen that 3D-C/C composites artificial rib has obvious capsule coverage, loose tissue around the capsule, clear muscle and fascia layers and no dense adhesion. In addition, there is no adhesion between 3D-C/C composites artificial rib and lung tissue in thoracic cavity (Supplementary Fig. S8c,d). After 3D-C/C composites artificial rib is implanted, there is obvious tissue growth inward and capsule formation. Moreover, the implantation does not cause obvious inflammatory reaction of surrounding soft tissues and dense adhesion.

**Figure 7:**
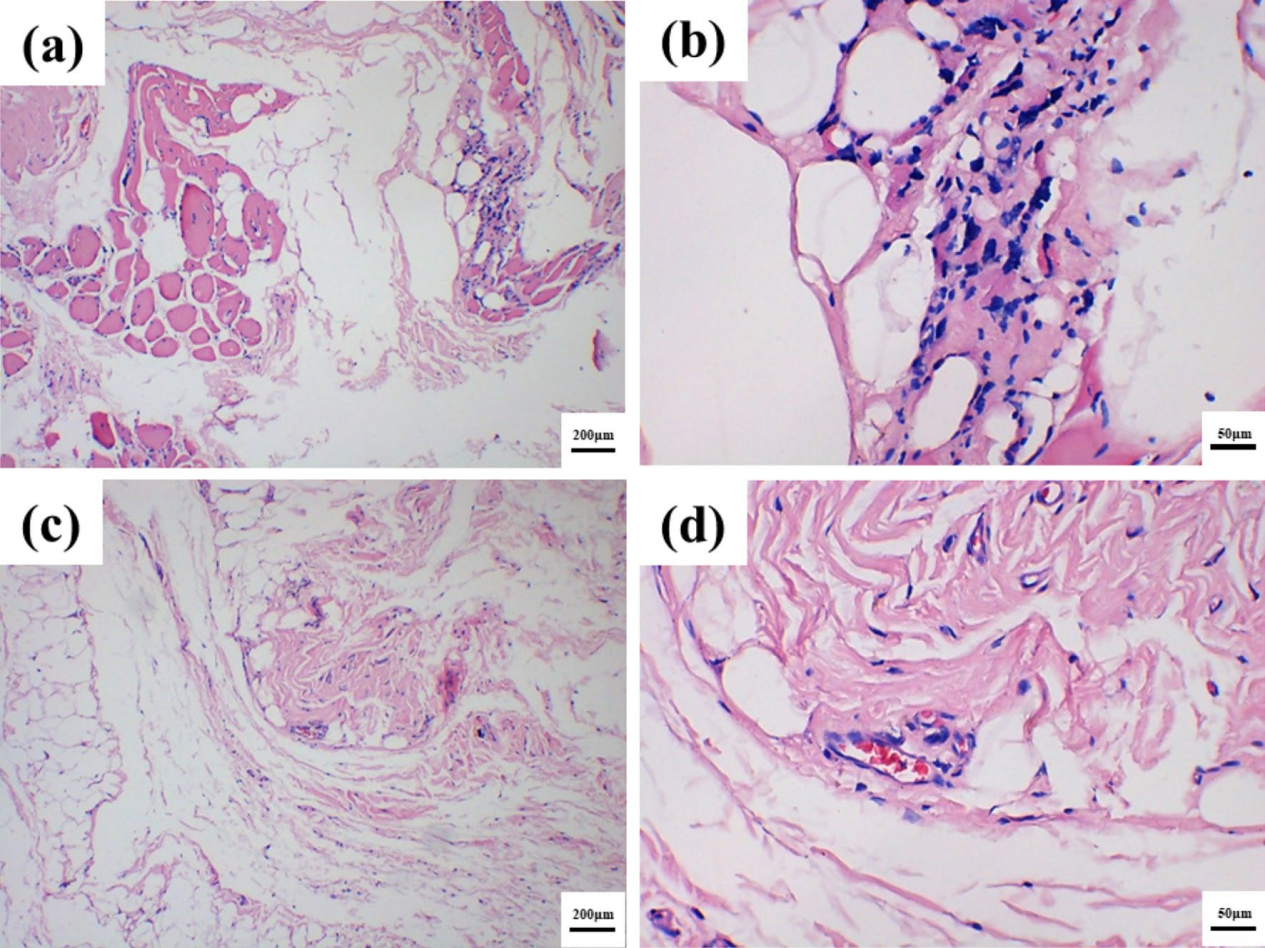
HE stained histological cross-sections of soft tissues of 3D-C/C composites artificial rib implantation after 1 year, joint between 3D-C/C composites artificial rib implant and ribs, (**a**) ×100, (**b**) ×400; 3D-C/C composites surface, (**c**) ×100, (**d**) ×400, respectively.

Figure 7 is a picture of soft tissue sections at 3D-C/C composites artificial rib/rib junction and 3D-C/C composites site after implantation in dogs for 1 year. There is no obvious hyperplasia and inflammatory reaction in soft tissue. The tissue is loose, the layers are clear, and there is no obvious vascular hyperplasia. It then could be concluded that 3D-C/C composites artificial rib is suitable for chest wall reconstruction.

## Conclusions

1. The synthesized 3D-C/C composites have a Young’s modulus of 5 GPa, a tensile strength of 167 MPa and an elongation of 5.0%. Those mechanical properties remain unchanged after fatigue (1 × 10^6^ cyclic tensile loading). Compared with 2.5D-C/C composites and TC4, 3D-C/C composites show mechanical properties close to autogenous bones, indicating a better biomechanical compatibility.
2. The composite structure of 3D-C/C composites enables a porosity of 16%, and 51% pores have a size larger than 100 μm. MG-63 cells can effectively adhere, spread and proliferate on the 3D-C/C composites surface as a result of the open pore structure. Besides, the CT imaging shows that 3D-C/C composites implants have no artifacts on imagery. This indicates 3D-C/C composites has good cell compatibility.
3. The artificial ribs made by 3D-C/C composites have been implanted into a large mammal dog. There is no obvious proliferation and inflammation in the soft tissues around the implant. Obvious tissue inward growth and capsule formation on the surface of 3D-C/C composites artificial rib have been observed after 1 year.

Above results have suggests that 3D-C/C composites artificial rib is suitable for chest wall reconstruction in comparison to the traditional 2.5D-C/C composites and TC4, and shed lights on future development of 3D-C/C composites for biological applications.

## Supplementary Information

Supplementary Information 1.
