# Supplementary material for: Synthesis, structure, and properties of carbon/carbon composites artificial rib for chest wall reconstruction
Source: Sci Rep. 2021 May 28;11:11285. doi: 10.1038/s41598-021-90951-8 (PMC8163812; doi:10.1038/s41598-021-90951-8)
Supplement: Supplementary file 1 — Supplementary Information 1. [file 41598_2021_90951_MOESM1_ESM.pdf]

**Supporting information:**

# Synthesis, structure, and properties of carbon/carbon composites artificial rib for chest wall reconstruction

Zhoujian Tan[1], Xiang Zhang[2], Jianming Ruan[1], Jiqiao Liao[2], Fenglei Yu[3], Lihong Xia[*4], Bin Wang[*3], Chaoping Liang[1]

[1]State Key Laboratory for Powder Metallurgy, Central South University, Changsha, Hunan Province, 410083, P. R. China

[2]Hunan Tankang Biotech Co., LTD., Changsha, Hunan Province, 410083, P. R. China

[3]Department of Thoracic Surgery, the Second Xiangya Hospital, Central South University, Changsha, Hunan Province, 410011, China. E-mail: wangbin23@csu.edu.cn

[4]College of Chemistry and Chemical Engineering, Central South University, Changsha, Hunan Province, 410083, P. R. China. E-mail: lihong_xia@csu.edu.cn

Table S1 Damage of C/C composites after impact testing.

| | 2.5D-C/C composites | 3D-C/C composites |
| --- | --- | --- |
| Before testing | 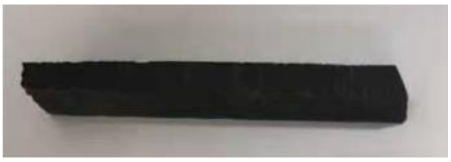 | 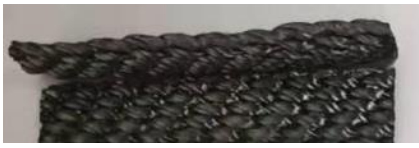 |
| After testing | 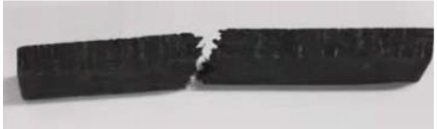 | 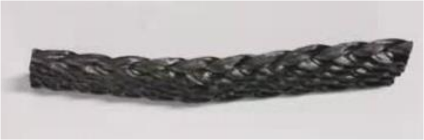 |

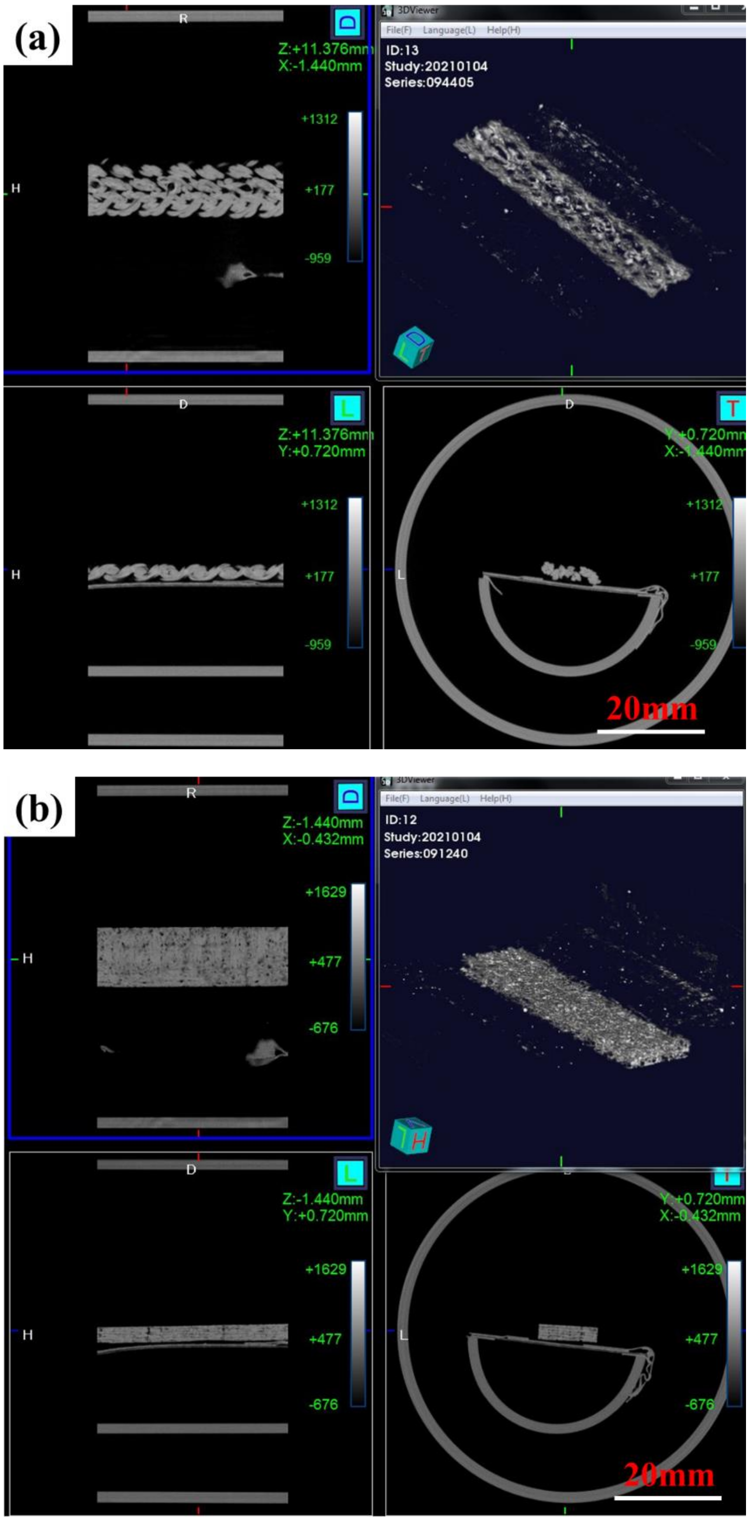


Fig. S1 Micro-CT of C/C composites, (a) 3D-C/C composites, (b) 2.5D-C/C composites, respectively.

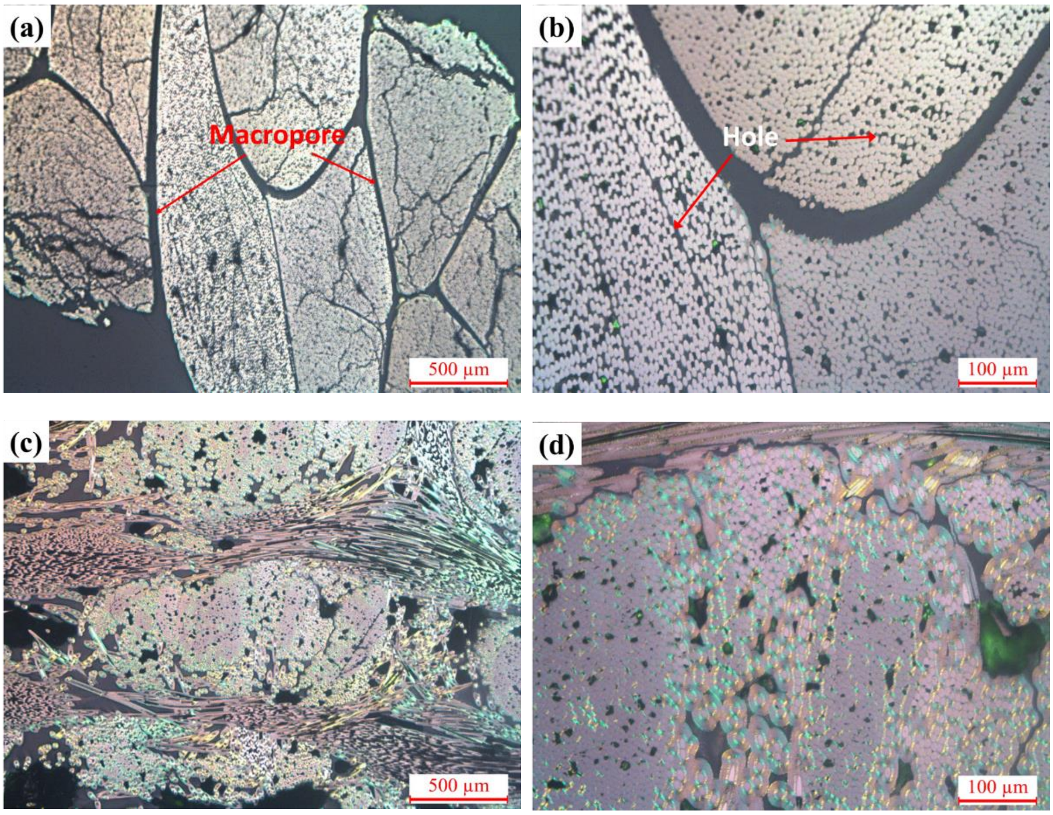


Fig. S2 Microstructure of C/C composites by polarized light microscope, (a), (b) for 3D-C/C composites; (c), (d) for 2.5D-C/C composites, respectively.

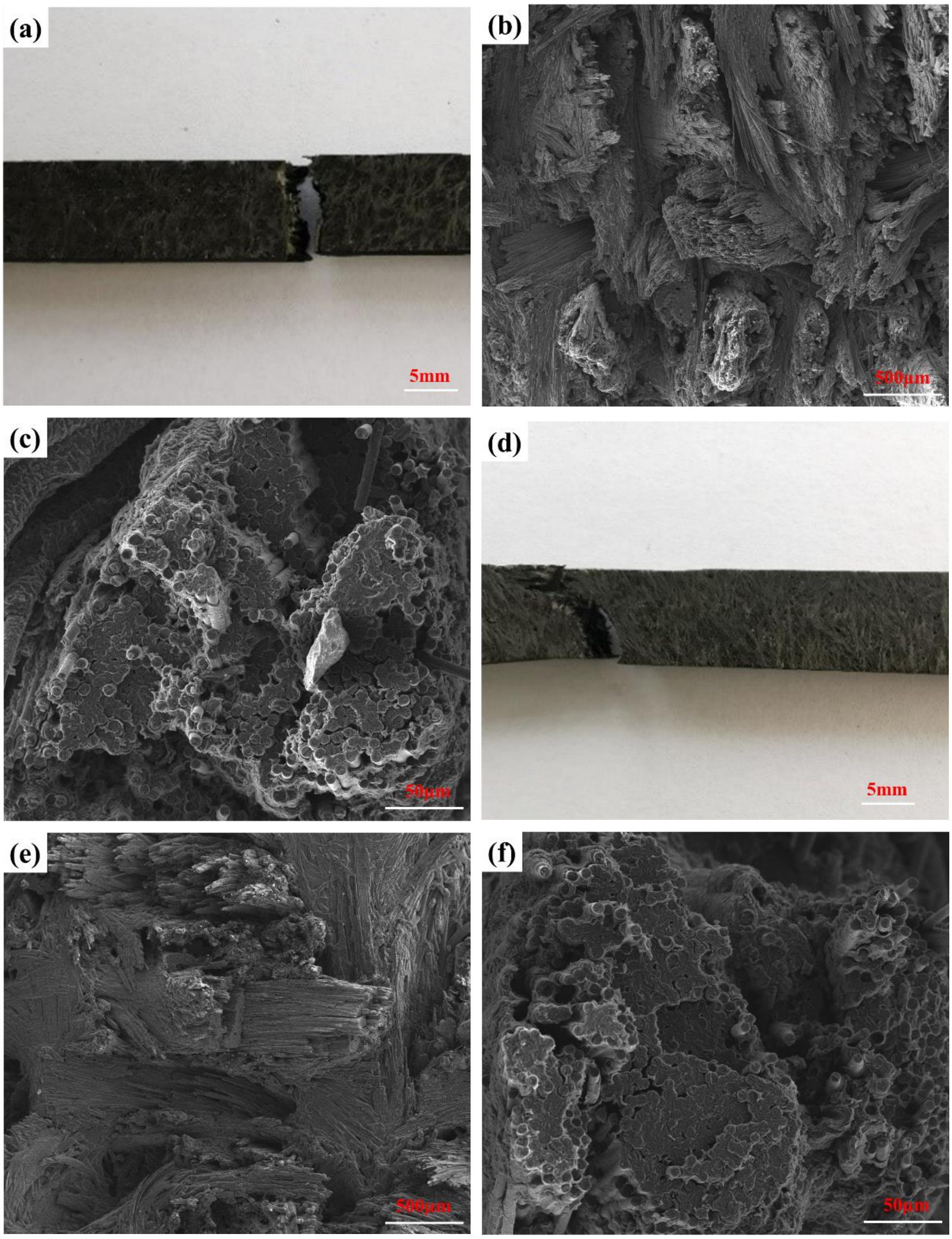


Fig. S3 Fractography of 2.5D-C/C composites after tensile testing, (a)~(c) under static condition; (d)~(f) after fatigue, respectively.

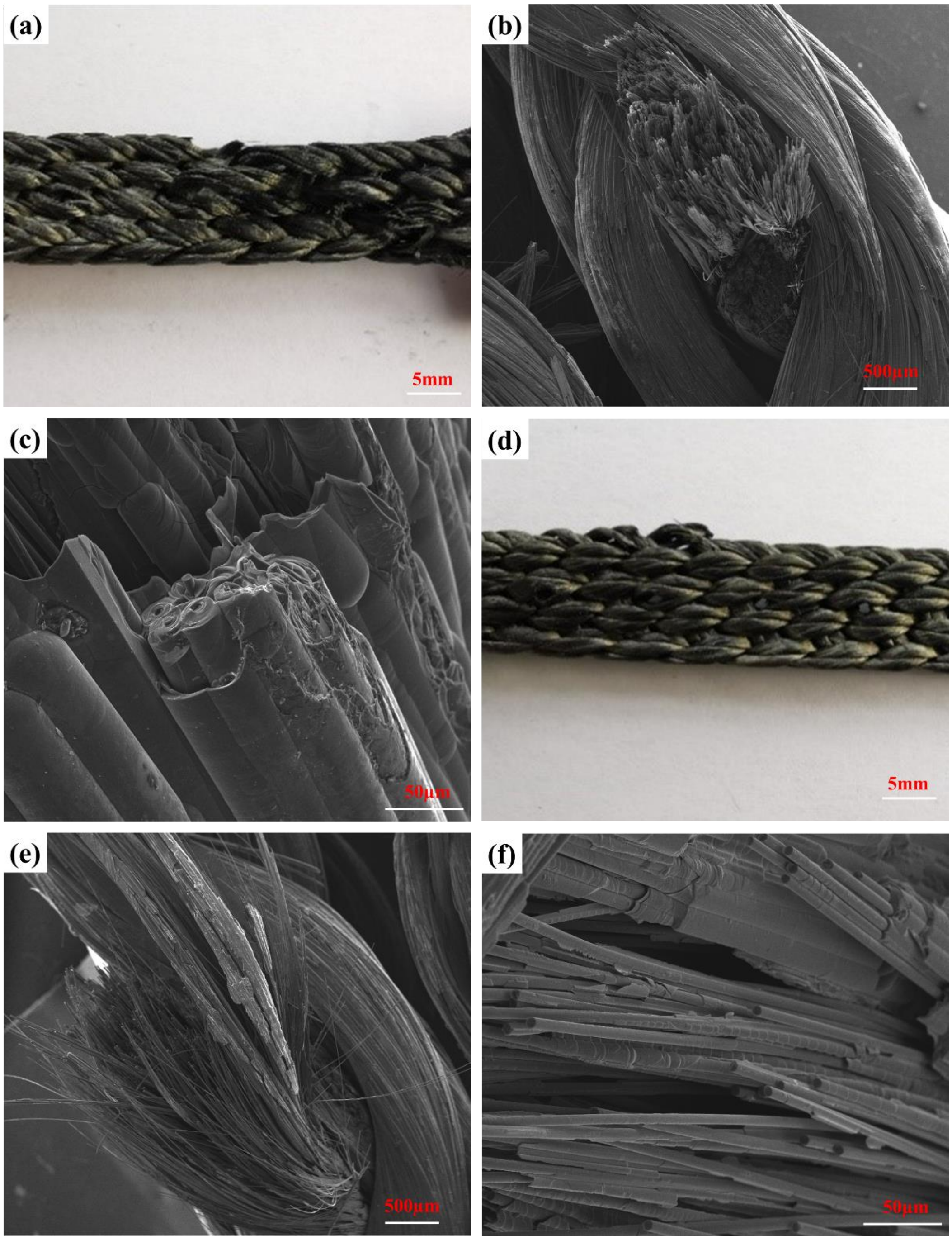


Fig. S4 Fractography of 3D-C/C composites after tensile testing, (a)~(c) under static condition; (d)~(f) after fatigue, respectively.

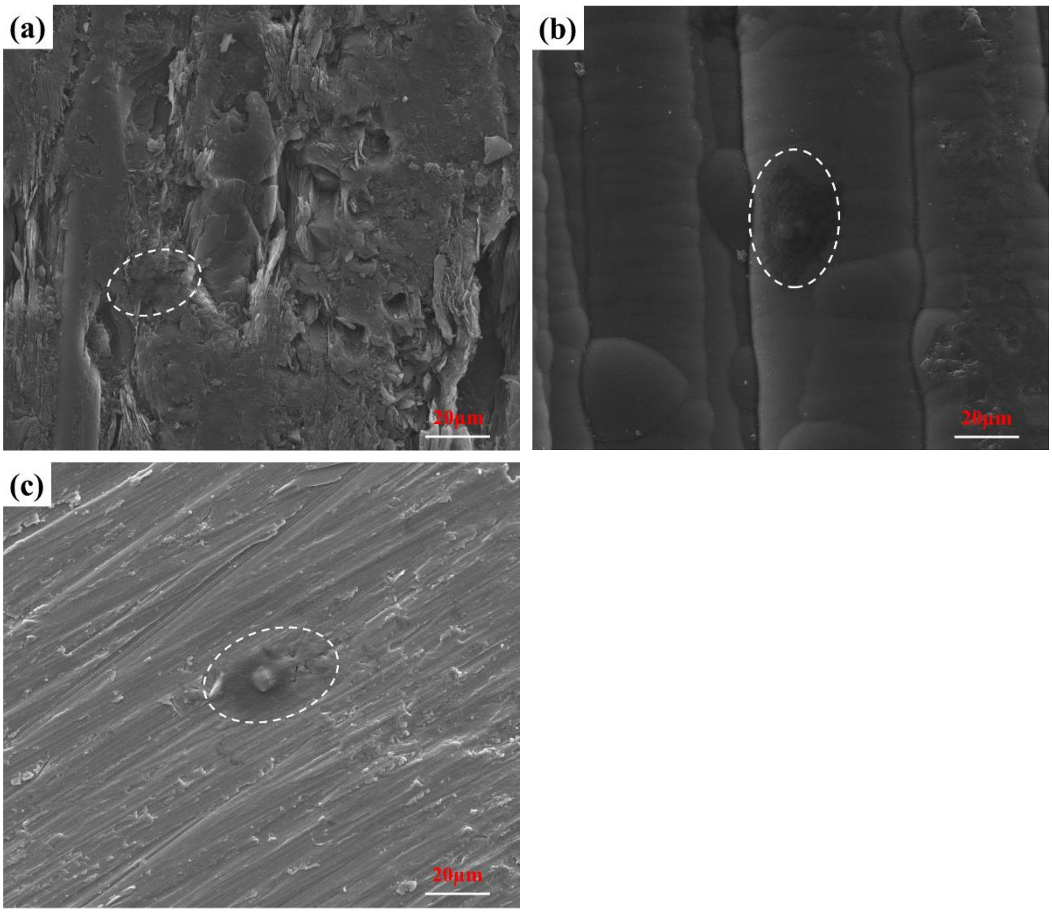


Fig. S5 SEM of MG-63 cells on materials surface in one day (MG-63 cell is in the dotted circle): (a) 2.5D-C/C composites; (b) 3D-C/C composites; (c)TC4, respectively.

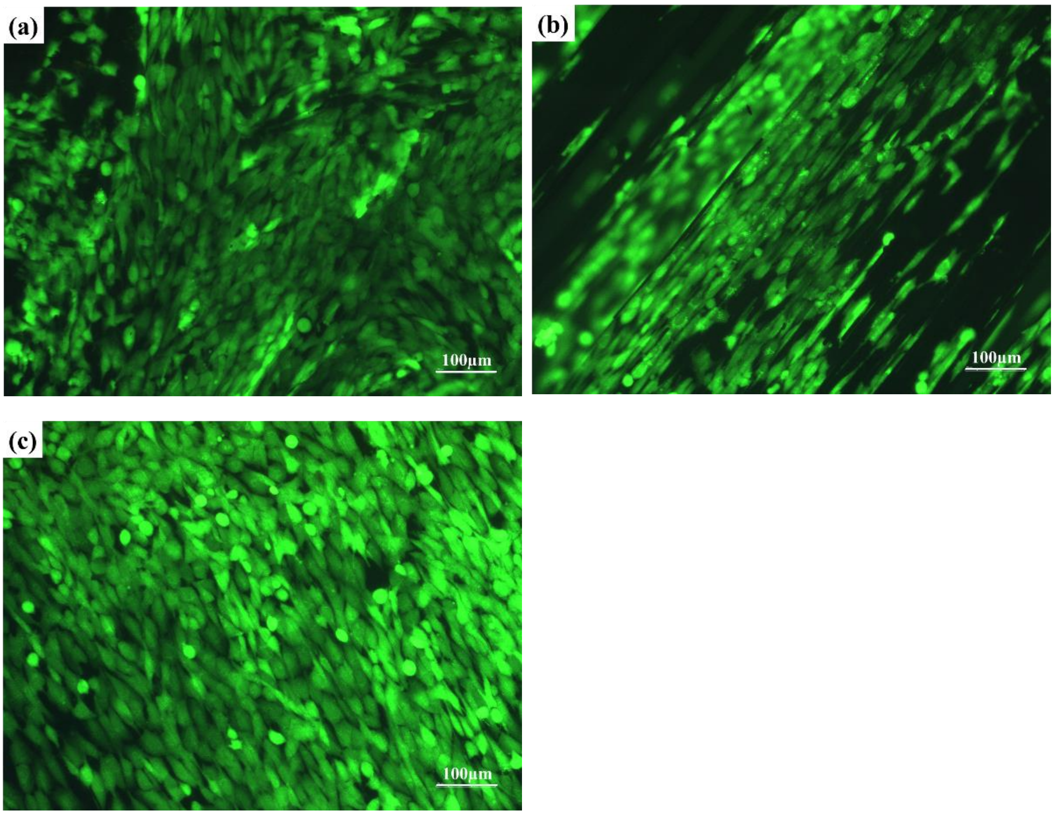


Fig. S6 FDA staining morphology of MG-63 cells on material surface in 5 days, (a) 2.5D-C/C; (b) 3D-C/C composites; (c)TC4, respectively.

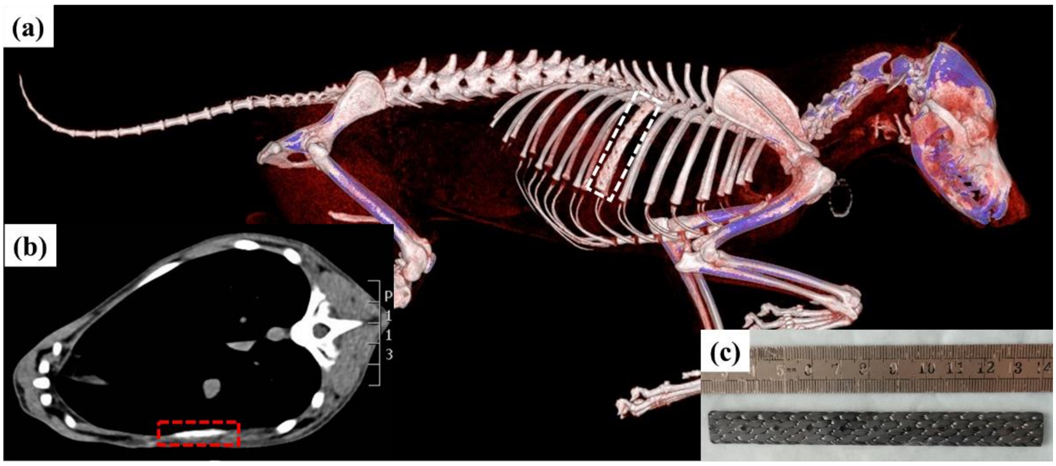


Fig. S7 CT images of 3D-C/C composites artificial ribs implanted in dogs (Sample is in the dotted circle), (a) three-dimensional reconstruction; (b) Fault scanning; (c) 3D-C/C composites artificial rib, respectively.

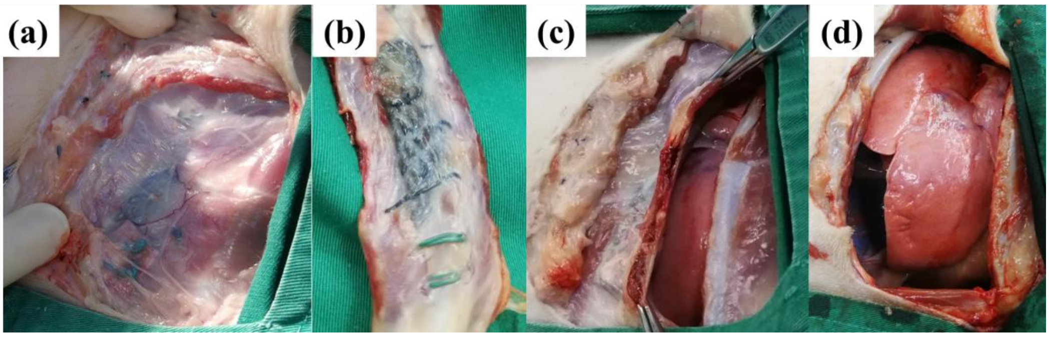


Fig. S8 Photograph of 3D-C/C composites artificial rib implantation after 1 year, (a), (b) 3D-C/C composites surface; (c), (d) 3D-C/C composites nearby tissue, respectively.
